# How to Eat Lemons

**Published:** 1881-08

**Authors:** 


### HOW TO EAT LEMONS.

Most people know the benefit of lemonade before breakfast, but few know how it is more than doubled by taking another at night, also. The way to get the better of a bilious system without blue pills or quinine is to take the juice of one, two or three lemons, as the appetite craves, in as much ice water as makes it pleasant to drink, without sugar, before going to bed. In the morning, on rising, or at least half an hour before breakfast, take the juice of one lemon in a goblet of water. This will clear the system of humors and bile, with mild efficacy, without any of the weakening effects of Congress water. People should not irritate the stomach by eating the lemons clear; the powerful acid of the juice, which is almost corrosive, infallibly produces inflammation after a while; but properly diluted, so that it does not burn or draw the throat, it does its full medicinal work without harm, and when the stomach is clear of food has abundant opportunity to work the system thoroughly.

### ONE GOOD DEED.

Bill Poole, of New York, sport and pugilist, whose murder by Lewis Baker years ago created such excitement, was not a model man. He did not pretend to be, and while so pretending rob his fellows. He had some generous traits which may be reckoned up hereafter. It is related of him that one Thanksgiving day, just before he was killed, he came to a marketman's stand and putting down $100 said : "Send chickens and turkeys for that amount to the charities." "You can't have all of this," the dealer replied. "We mean to have a hand in." The result was the poultry dealers of the market clubbed together, and the next day all the principal charities of New York received turkeys and chickens without stint. Bill Poole's $100 was only a drop in the bucket, but it was the drop that filled it. Since that time Washington Market has contributed tons of poultry and meat to the charities of the city, and has organized a system that aids materially in the efforts of the charitable to make at least one day of the year a pleasant and happy one to the unfortunate.

This shows how one good action often impels many, and it demonstrates too that, among the outcast of the earth, there is often a flicker of the divine spark that redeems them from total depravity.

Harness Blacking.—In an old scrap book I find this recipe which is said "will give a beautiful polish :" 2 ozs. white wax and 3 ozs. of turpentine are to be dissolved together over a slow fire; then add 1 oz. ivory-black and 1 drachm indigo, to be well pulverized and mixed together. "When the wax and turpentine are dissolved, add the ivory-black and the indigo, and stir till cold." Apply very thin and brush afterwards.

A New York paper speaks of a man who was "beaten in three suits," which reminds one of the old-time schoolboy who used to pad his trousers in anticipation of a thrashing.

